# Characteristics and Influencing Factors Among Newly Diagnosed HIV-1 Patients with Non-Marital, Non-Commercial Heterosexual Contact in Lishui, China (2020–2024)

**DOI:** 10.3390/v17121626

**Published:** 2025-12-16

**Authors:** Jianhua Mei, Jinkai Li, Xiaolei Chen, Liyang Qiu, Haifang Zhang, Jie Yu, Ling Ye, Deyong Zhang, Dongqing Cheng, Xiuying Chen

**Affiliations:** 1 Lishui Center for Disease Control and Prevention, Lishui 323000, China; 2588640@163.com (J.M.); cxl5771@163.com (X.C.); qljly@foxmail.com (L.Q.); hfz525@163.com (H.Z.); yj133213@outlook.com (J.Y.); 2 School of Medical Technology and Information Engineering, Zhejiang Chinese Medical University, Hangzhou 310053, China; jinkai200077@163.com; 3 Hangzhou Center for Disease Control and Prevention, Hangzhou Health Supervision Institution, Hangzhou 310021, China; lqyeling1986@outlook.com

**Keywords:** HIV-1, non-marital non-commercial heterosexual contact, transmitted drug resistance, molecular transmission network

## Abstract

The increasing proportion of HIV-1 infections transmitted via non-marital non-commercial heterosexual contact (NMNCHC) in China necessitates a deeper understanding of its local characteristics. This study investigated the epidemiological, molecular network, and drug-resistant profiles among 400 newly diagnosed HIV-1 patients infected via non-marital heterosexual contact (NMHC), specifically its non-commercial subtype, in Lishui from 2020–2024. HIV-1 pol gene sequences were analyzed for subtypes, drug resistance mutations, and transmission clusters using phylogenetic and network methods (genetic distance threshold: 0.9%). The overall prevalence of transmitted drug resistance (TDR) was 13.3%, an intermediate level exceeding the national average, driven predominantly by NNRTI resistance (6.3%). High-level resistance to NVP (3.0%) and EFV (2.75%) was observed. CRF08_BC (43.8%) was the dominant subtype. Multivariate analysis identified female gender and higher education as significant risk factors for NMNCHC acquisition. Molecular network analysis incorporated 55.3% of cases, revealing clusters predominantly composed of middle-aged and elderly males, with CRF08_BC and CRF01_AE showing higher NMNCHC transmission risk within networks. These findings underscore an evolving epidemic with significant TDR and highlight the urgent need for targeted interventions, including enhanced resistance surveillance and focused strategies for the concealed NMNCHC population, to curb local HIV-1 transmission.

## 1. Introduction

AIDS remains a pressing global public health concern, despite the exhaustive global efforts dedicated to curbing HIV-1 transmission [1]. As reported by UNAIDS, the year 2024 saw 40.8 million individuals living with HIV globally, while 630,000 deaths were linked to related illnesses. Furthermore, globally, since the start of the epidemic, approximately 91.4 million people have been infected by HIV, while AIDS-related conditions have claimed an estimated 44.1 million lives worldwide [2].

The primary transmission routes during the early phase of the AIDS epidemic in China were drug injection and blood exposure [3,4]. With the enactment and implementation of relevant laws and regulations [5,6], the likelihood of transmission via these two routes has significantly decreased [7], having been superseded by sexual contact transmission [8]. Among these, heterosexual transmission constitutes the primary route for newly diagnosed HIV-1 infections in China each year, with its proportion steadily increasing from 8.7% in 2008 to 73.4% in 2024 [9,10].

According to the marital status of HIV patients, heterosexual transmission can broadly be categorized into two groups: non-marital transmission and marital transmission. Non-marital transmission exhibits a more widespread distribution, possessing greater social and concealed characteristics compared to HIV transmission within marriage, thereby presenting greater challenges for prevention and control [11]. Since 2014, commercial/non-commercial contact history labels have been included in the Comprehensive Response Information Management System (CRIMS) to better differentiate heterosexual transmission forms, subsequently enabling further division of non-marital heterosexual transmission into two key categories.

Previous domestic and international research has predominantly focused on HIV-single-positive spouses within marriages and populations engaging in commercial heterosexual intercourse, with relatively little attention paid to those involved in non-marital non-commercial heterosexual contact (NMNCHC) [12,13]. Compared to HIV transmission through NMNCHC within high-risk populations, transmission via such contact within the general population is more concealed and difficult to monitor using traditional methods, thereby posing greater challenges for prevention and control. In China, the proportion of HIV infections transmitted through NMNCHC has risen year on year, which makes this group one that cannot be overlooked in AIDS prevention and control efforts [14].

Moreover, drug resistance represents an increasingly serious global health concern. As antiretroviral therapy progresses, HIV-1 accumulates drug-resistant mutations, diminishing its sensitivity to ART drugs and ultimately developing resistance [15]. One of the WHO studies indicates that the number of countries where the prevalence of transmitted resistance to non-nucleoside reverse transcriptase inhibitors (NNRTIs) exceeds the 10% threshold is increasing exponentially [16]. This underscores the importance of a comprehensive understanding of HIV transmission and drug resistance mechanisms, which can guide the enhancement of existing treatment policies [17].

Previous surveillance data and studies indicate that heterosexual transmission constitutes the primary route of infection in Zhejiang Province, such as Ningbo, Wenzhou, and Lishui. However, analyses of characteristics and Influencing Factors among HIV-1 cases with NMNCHC remain scarce. Consequently, it is imperative to conduct a detailed characterization of HIV-1 infections transmitted through NMNCHC in Lishui to enhance our understanding of the AIDS epidemic [18,19,20].

This study aims to analyze the characteristics and influencing factors among ART-naïve HIV-1 patients who were infected via non-marital heterosexual contact (NMHC), specifically through its non-commercial subtype, in Lishui from 2020 to 2024, providing recommendations and scientific evidence for targeted HIV prevention and control efforts.

## 2. Materials and Methods

### 2.1. Study Design and Data Collection

This study retrospectively gathered demographic, clinical data, and HIV subtype information from 400 ART-naïve individuals with non-marital heterosexual contact (NMHC) in Lishui, China, between January 2020 and December 2024. To clarify the core terminology and eliminate classification ambiguity, three subtypes of sexual contact involved in this study were explicitly defined:

Non-marital heterosexual contact (NMHC): Refers to all heterosexual sexual contacts occurring outside legal marital relation-ships, covering both commercial and non-commercial subtypes, excluding marital and same-sex sexual contacts.

Non-marital commercial heterosexual contact (NMCHC): A commercial subtype of NMHC, specifically referring to sexual contacts between non-spousal heterosexuals for monetary or material rewards, including paid sexual ser-vices and interest-exchange sexual contact scenarios.

Non-marital non-commercial heterosexual contact (NMNCHC): A non-commercial subtype of NMHC, denoting non-spousal heterosexual sexual contacts without monetary or material exchange, which may involve occasional or sus-tained contacts with specific partners or temporary contacts with unstable relationships, excluding all NMHC-related contacts with commercial interests.

The inclusion criteria were as follows: (1) the newly diagnosed place is Lishui; (2) the date of diagnosis was between 1 January 2020 and 31 December 2024; (3) the contact history was NMCHC and NMNCHC; (4) had never received treatment. After obtaining informed consent from all participants, demographic information was collected.

### 2.2. Laboratory Methods

Newly confirmed HIV-positive cases are invited to be part of the study at the time of notification. Research blood samples were collected concurrently with CD4 testing, and the collection sites were local Centers for Disease Control and Prevention or medical institutions. The medical teams were designated HIV treatment facilities in county-level and above general hospitals in various regions. Samples were stored at −80 °C, and HIV detection was performed in line with national protocols. Detailed information regarding national standard [21] for HIV and CD4 cell testing could be obtained through official websites such as the National Health Commission of the People’s Republic of China. HIV-1 RNA was extracted from plasma samples using the Viral RNA Mini Kit (Tianlong, Suzhou, China) and supporting kits in line with the manufacturer’s instructions. The RNA was subsequently amplified by an in-house nested reverse transcription PCR protocol targeting the HIV-1 pol gene region (1316 bp; HXB2: 2147–3462) [22]. PCR products were confirmed by 1% agarose gel electrophoresis, and positive amplicons were purified and subjected to Sanger sequencing by Hangzhou, China Qingke Biotechnology Co., Ltd.

### 2.3. Sequence Analysis and Identification of Genotypes

Sequence fragments were assembled and calibrated using Sequencer 5.4.6 (Gene Codes, Ann Arbor, MI, USA), followed by manual alignment and editing in BioEdit 7.7.1. All sequences were compared against reference sequences from the HIV Database. A neighbor-joining phylogenetic tree was constructed in MEGA 6.0 [23]. Circulating recombinant forms (CRFs) were identified by using the COMET tool [24] (https://comet.lih.lu, accessed on 17 September 2025). Unclassified recombinant forms (URFs) were analyzed for recombination patterns using the RIP 3.0 and jpHMM (updated on 11 February 2015) online tools.

### 2.4. Analysis of Drug-Resistance Mutations

Aligned sequences were submitted to the Stanford HIV Drug Resistance Database for the analysis of drug resistance mutations (DRMs) using the HIVdb program (version 9.8). The resulting mutations were evaluated according to the Stanford Penalty Score, which classifies resistance as low (score 15–29), intermediate (30–59), or high (≥60) against a series of drugs: 7 NRTIs [lamivudine (3TC), abacavir (ABC), tenofovir (TDF), zidovudine (AZT), emtricitabine (FTC), stavudine (D4T), didanosine (DDI)]; 5 NNRTIs [efavirenz (EFV), rilpivirine (RPV), etravirine (ETR), nevirapine (NVP), doravirine (DOR)]; and 8 PIs [nelfinavir (NFV), tipranavir/r (TPV/r), indinavir/r (IDV/r), fosamprenavir/r (FPV/r), darunavir/r (DRV/r), saquinavir/r (SQV/r), lopinavir/r (LPV/r), atazanavir/r (ATV/r)].

### 2.5. Construction of Genetic Transmission Networks

Molecular transmission networks were analyzed using a Genetic distance (GD) threshold of 0.009, which was identified as optimal for maximizing the detection of transmission clusters (TCs). Genetic distances were computed in MEGA 6.0 under the Tamura-Nei model, and network visualization was performed with Cytoscape 3.9.1 [25]. Network topology was interpreted where nodes denote sequences, edges represent transmission links, and degree reflects connection count. According to prior definitions, cases were classified as non-clustered (degree = 0), clustered (degree ≥ 1), low-risk (degree ≤ 3), or high-risk (degree ≥ 4). The proportion of sequences forming the network defined the network entry rate [25].

### 2.6. Statistical Analyses

Data management and statistical analysis were performed using EpiData 3.1, SPSS Statistics 26.0 (IBM, Armonk, NY, USA), and office software. Demographic and genotypic characteristics were compared by chi-square or Fisher’s exact tests, with the latter applied for small sample sizes (*n* < 40) or expected frequencies below 5. Determinants of molecular network inclusion were assessed by univariate and multivariate logistic regression. Statistical significance was defined as *p* < 0.05.

## 3. Results

### 3.1. Demographic Characteristics of the Study Population

Among persons with HIV-1 infected by NMHC from 2020 to 2024, 400 (95.2%) were successfully sequenced and constituted the study cohort. The cohort was predominantly of Han ethnicity (94.8%, 379/400), male (72.5%, 290/400), and farmer (63.7%, 255/400). The majority (52.3%, 238/400) were aged 50 years or older, with a mean age of 52.5 years. Most participants were married (51.0%, 204/400) and had an educational attainment of elementary school (55.5%, 222/400) or junior middle school (32.5%, 130/400). Moreover, the main subtype of infection was CRF08_BC (43.8%, 175/400) (Table 1).

**Figure 1:**
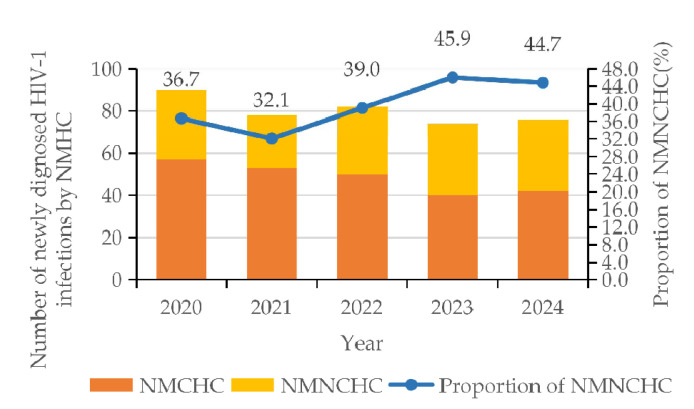
Trends in newly diagnosed HIV-1 patients with NMCHC and NMNCHC from 2020 to 2024.

The number of HIV infections transmitted through NMCHC decreased from 57 cases in 2020 to 40 cases in 2023, subsequently rising to 42 cases in 2024. The proportion of HIV infections transmitted through NMNCHC increased from 32.1% in 2021 to 45.9% in 2023, before declining slightly to 44.7% in 2024, exhibiting an overall upward trend (Figure 1). χ^2^ test revealed that there was a significant relationship between HIV infection (among individuals infected through NMNCHC) and gender (*p* < 0.001), ethnicity (*p* = 0.002), age (*p* < 0.001), educational attainment (*p* = 0.038), occupation (*p* = 0.039), and HIV-1 subtype (*p* = 0.001). Multivariate analysis revealed that females (95% CI = 10.796–36.975, *p* < 0.001) were 19.980 times more likely to become infected with HIV through NMNCHC; those with senior high school education (95% CI = 1.661–11.118, *p* = 0.003) and junior college or above education (95% CI = 1.417–15.776, *p* = 0.012) were 4.30 and 4.73 times more likely to become infected with HIV through NMNCHC, respectively; those with CRF08_BC (95% CI = 0.239–0.940, *p* = 0.033) were 0.475 times more likely to be-come infected with HIV through NMNCHC (Table 1).

### 3.2. Subtype Analysis

**Figure 2:**
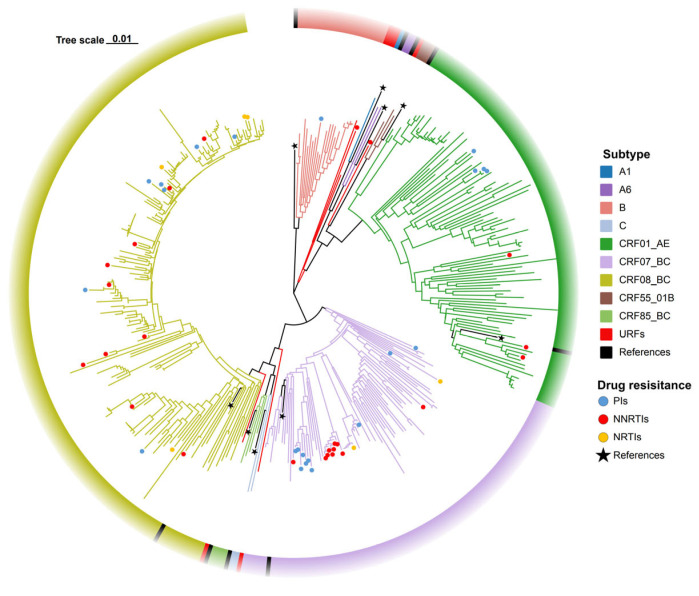
Phylogenetic tree of ART-naïve HIV-1 patients with NMHC between 2020 and 2024.

Genotypic analysis of 400 successfully amplified samples identified 13 distinct subtypes, comprising 9 CRFs and 3 URFs. The CRF distribution was as follows: CRF08_BC (43.8%, 175/400), CRF07_BC (22.3%, 89/400), and CRF01_AE (24.0%, 96/400) were predominant, followed by subtype B (5.5%, 22/400), CRF85_BC (1.0%, 4/400), CRF55_01B (0.8%, 3/400), A6 (0.5%, 2/400), subtype C (0.5%, 2/400), and A1 (0.25%, 1/400). Among URFs, four were CRF07_BC/CRF01_AE (1.0%), one was B/C (0.25%), and one was CRF01_AE/B (0.25%) (Figure 2). Additionally, drug resistance was predominantly observed in the CRF07_BC (5.5%, 22/400) and CRF08_BC (5.3%, 21/400), with NNRTIs exhibiting resistance rates of 40.0% (10/25) and 40.0% (10/25) in these subtypes, respectively, NRTIs at 33.3% (2/6) and 66.7% (4/6), and PIs at 45.5% (10/22) and 31.8% (7/22) (Figure 2).

### 3.3. Drug Resistance Analysis

**Figure 3:**
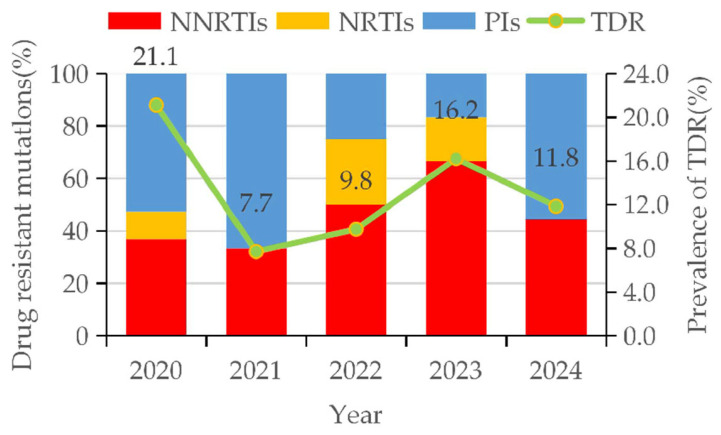
Trends in drug-resistant mutations and prevalence of TDR among HIV-1 patients with NMHC.

Between 2020 and 2024, the overall prevalence of transmitted drug resistance (TDR) was 13.3% (53/400). By drug class, NNRTI resistance was the most common (6.3%, 25/400), followed by PI resistance (5.8%, 23/400) and NRTI resistance (1.5%, 6/400). A marked increasing trend was observed in TDR prevalence, rising from 7.7% in 2021 to 16.2% in 2023. This rise was primarily driven by NNRTI resistance, which increased from 2.6% to 10.8% over the same period and constituted a growing proportion of TDR cases (from 33.3% to 66.7%). In contrast, PI resistance declined from 11.1% in 2020 to 2.4% in 2023, and its contribution to overall TDR fell from 66.7% to 16.7% between 2021 and 2023 (Figure 3).

**Figure 4:**
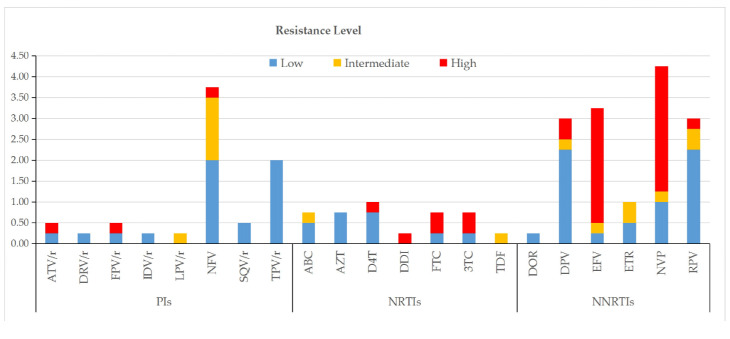
Drug resistance levels among ART-naïve HIV-1 patients with NMHC.

According to the Stanford HIVdb algorithm, the prevalence of TDR to single agents was highest for NVP (4.3%, 17/400), NFV (3.8%, 15/400), EFV (3.3%, 13/400), RPV (3.0%, 12/400), and TPV/r (3.0%, 12/400), while the lowest rate (0.3%, 1/400) was shared by DRV/r, IDV/r, LPV/r, DDI, TDF, and DOR. At the drug class level, TDR was most common against NNRTIs (14.8%, 59/400), followed by PIs (8.0%, 32/400) and NRTIs (4.5%, 18/400). High-level resistance was most frequently observed for NVP among NNRTIs (3.0%, 12/400), for ATV/r, FPV/r, and NFV among PIs (each 0.3%, 1/400), and for FTC and 3TC among NRTIs (each 0.5%, 2/400) (Figure 4).

### 3.4. Molecular Transmission Network Analysis

**Figure 5:**
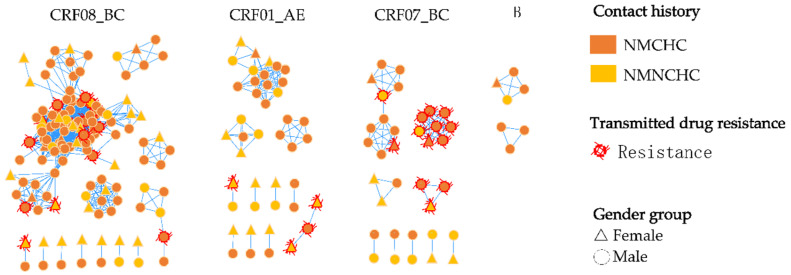
Characterization of the molecular network in ART-naïve HIV-1 patients with NMCHC and NMNCHC.

In total, 55.3% (221/400) of patients were recruited into the molecular network at a genetic distance (GD) threshold of 0.9%, forming 34 distinct transmission clusters. The cluster sizes ranged from 2 to 96, with a median of 6.5; 19 clusters comprised only 2 members, while a single large cluster included 96 individuals. Among the 221 cases within the network, 76.9% (170/221) were male. Furthermore, CRF08_BC was the predominant subtype, constituting 62.0% (137/221) of cases, followed by CRF01_AE (19.0%, 42/221), CRF07_BC (15.8%, 35/221), and subtype B (3.2%, 7/221). Regarding the history of contact, 31.2% (69/221) were infected through NMNCHC, while 68.8% (69/221) were infected through NMCHC. Within the network of B sequences, 83.3% (5/6) were infected through NMCHC. The predominant resistance mutation within this network was S68G (21.2%, 14/66), followed by V179D (18.2%, 12/66), K103N (12.1%, 8/66), and M46I (6.1%, 4/66) (Figure 5).

## 4. Discussion

In this study, we investigated the demographic and spatial distribution characteristics of newly diagnosed HIV patients in Lishui, and further analyzed potential factors associated with HIV infections among the population infected via non-marital heterosexual contact, specifically its non-commercial subtype.

This indicated that non-marital heterosexual contact plays a significant role in HIV transmission, while also demonstrating the effectiveness of the Chinese government’s intervention policies targeting high-risk populations such as the injecting drug population and MSM [26]. Among these, infections stemming from non-commercial heterosexual contact accounted for 39.5% (158/400), lower than Anhui Province’s 48.3% [27] and Sichuan Province’s 41.3% [28]. However, this figure exhibited an overall upward trend, rising from 32.1% in 2021 to 44.7% in 2024, which suggests that when formulating HIV prevention and control measures, particular attention should be paid to the NMNCHC population.

Moreover, in this investigation, women exhibited a lower probability of acquiring HIV through heterosexual contact compared to men, at merely 27.5% (110/400), which suggests that HIV infection patterns in Lishui exhibit distinct regional characteristics. We postulate that the mountainous geography of the region acts as a key contributing factor. This topography restricts external communication, consistent with the finding that 91.8% of HIV-infected individuals in this study currently reside in Lishui, resulting in a more stable population structure. Consequently, these conditions foster localized epidemic dynamics. This study found that individuals with a marital status of married (35.7, 74/207) were less likely to contract HIV through NMNCHC compared to those who were unmarried (38.6, 32/83) or divorced/widowed, which was contrary to the results of the Dehong study [29]. This may be attributed to singles tending to be more susceptible to the influence of their social circles and possess a stronger sense of curiosity, which in turn increases their risk of infecting HIV through NMNCHC. Furthermore, additional research indicated that women tend to be more committed in intimate relationships. In contrast, men were more inclined to seek companionship from others to alleviate their sense of loneliness, which means men faced a significantly higher probability of contracting HIV through NMCHC than through NMNCHC [14]. This aligns with the findings of this study, where 76.9% (223/290) of men acquired HIV through NMCHC, compared to 19.1% (21/110) of women.

Inadequate HIV detection and delayed diagnosis continue to impede the global HIV response. The initial CD4+ T-cell count reflects the immune function level in HIV-infected individuals. When the T-cell count falls below 200 cells/μL, it indicates that the patient has entered the stage of severe immunodeficiency [30]. In this study, 36.3% of individuals had CD4+ T-cell counts below 200 cells/μL, indicating that at current testing coverage levels, a significant number of patients remain undetected during the early stages. Particularly as most elderly individuals have underlying health conditions, symptoms of HIV infection are often masked, leading to a prolonged interval between infection and diagnosis. This may result in missed opportunities for early detection. To address this challenge, alongside conventional prevention and control measures, innovative testing models are required to enhance public knowledge about HIV/AIDS and encourage proactive testing awareness.

Results from the multivariate logistic regression analysis demonstrated that females (OR = 19.980, *p* < 0.001), individuals with a Senior high school education (OR = 4.297, *p* = 0.003), and those with a junior college or above education (OR = 4.727, *p* = 0.012) are more likely to contract HIV through NMNCHC. In the digital era, individuals with higher education often possess the technical proficiency to leverage dating platforms and online information, which may expedite partner seeking and increase engagement in unsafe sexual practices [31]. This underscores that future AIDS prevention and control efforts should account for the concealed nature of infections among individuals engaging in NMNCHC. Emphasis should be placed on strengthening intervention and control measures specifically targeting women living with HIV, alongside the targeted use of the internet to disseminate AIDS awareness and health education.

ART has fundamentally improved the prognosis of HIV by significantly reducing associated incidence and mortality, thereby transforming a once uniformly fatal infection into a manageable chronic condition and substantially prolonging survival, which stands in stark contrast to the rapid progression to AIDS within about 10 years without treatment [32,33]. However, a rapid increase in the prevalence of TDR has been observed recently, largely driven by the widespread lack of PDR testing. The prevalence of transmitted drug resistance (TDR) in Lishui was 13.3% (53/400), a rate that exceeded the national average (7.4%) [34] and qualified as intermediate by WHO standards (5–15%) [35]. The additional finding of high-level resistance to key first-line drugs NVP (3.0%, 12/400) and EFV (2.75%, 11/400) highlights the critical need for surveillance of these mutation hotspots to contain TDR [36].

The analysis of HIV molecular transmission networks plays a critical role in guiding precise interventions and optimizing the allocation of public health resources [25]. Unlike other cities with younger populations, such as Shenzhen [37] and Hangzhou [38], HIV-infected individuals within Lishui’s molecular transmission network predominantly consist of middle-aged and elderly individuals (75.1%, 166/221), with males accounting for 81.9% (136/166). The primary exposure history was NMCHC (88.2%, 120/136). This suggests that NMCHC among middle-aged and elderly men is a key driver of clustered transmission within the Lishui HIV network. It was also observed that the predominant HIV subtype transmitted through NMNCHC within the network was CRF08_BC (52.2%, 36/69). Furthermore, nearly half of the CRF01_AE subtype cases (47.6%, 20/42) were infected with HIV via NMNCHC. This shows that CRF08_BC and CRF01_AE carry a higher risk of transmission through NMNCHC.

This study has a few limitations. Firstly, as this investigation was conducted within a single city in southeastern China, the generalizability of our findings to other regions or countries may be limited. Secondly, potential inaccuracies may exist in the classification of contact history, which typically relies on self-reported accounts from infected individuals. Some of them, considering ethical factors among others, may misreport NMCHC as NMMCHC. Furthermore, while this study comprises 400 ART-naïve HIV-1 patients in Lishui (2020–2024), the voluntary basis of HIV testing could lead to selection bias, which may impair the sample’s representativeness. Finally, the genetic analysis was limited to the pol gene, targeting protease and reverse transcriptase mutations, and consequently precluded the assessment of resistance associated with INSTIs.

## Figures and Tables

**Table 1:** Characteristics of ART-naïve HIV-1 patients with NMHC in Lishui between 2020 and 2024.

Characteristics	Total *n* (%)	Univariate Analysis			Multivariate Analysis	
		Non-Marital Non-Commercial *n* (%)	χ^2^	*p*-Value	OR (95% CI)	*p*-Value
Total	400	158				
Gender			118.636	<0.001		
Male	290 (72.5)	67 (42.4)			1.000	
Female	110 (27.5)	91 (57.6)			19.980 (10.796–36.975)	< 0.001
Ethnicity			9.455	0.002		
Han	379 (94.8)	143 (90.5)			1.000	
Others	21 (5.3)	15 (9.5)			2.452 (0.727–8.264)	0.148
Current address						
Lishui	367 (91.8)	146 (92.4)	0.148	0.700		
Other cities	33 (8.3)	12 (7.6)				
Age (years)			13.556	<0.001		
<50	146 (36.5)	75 (47.5)			1.000	
≥50	254 (63.5)	83 (52.5)			0.784 (0.432–1.421)	0.422
Marital status			2.928	0.231		
Unmarried	83 (36.5)	32 (20.3)				
Married	204 (51.0)	74 (46.8)				
Divorced or widowed	113 (28.2)	52 (32.9)				
Education level			8.452	0.038		
Primary school or below	222 (55.5)	81 (51.3)			1.000	
Junior middle school	130 (32.5)	49 (31.0)			1.268 (0.692–2.323)	0.443
Senior high school	29 (7.2)	16 (10.1)			4.297 (1.661–11.118)	0.003
Junior college or above	19 (4.8)	12 (7.6)			4.727 (1.417–15.776)	0.012
Occupation			4.281	0.039		
Farmer	255 (63.7)	91 (57.6)			1.000	
Others	145 (36.3)	67 (42.4)			0.816 (0.453–1.470)	0.498
CD4, cells/ul			1.576	0.455		
<200	145 (36.3)	58 (36.7)				
≥200	254 (63.5)	99 (62.7)				
Unknow	1 (0.3)	1 (0.6)				
Year			4.280	0.369		
2020	90 (22.5)	33 (20.9)				
2021	78 (19.5)	25 (15.8)				
2022	82 (20.5)	32 (20.3)				
2023	74 (18.5)	34 (21.5)				
2024	76 (19.0)	34 (21.5)				
HIV-1 subtypes			15.557	0.001		
CRF07_BC	89 (22.3)	43 (27.2)			1.000	
CRF08_BC	175 (43.8)	50 (31.6)			0.475 (0.239–0.940)	0.033
CRF01_AE	96 (24.0)	46 (24.1)			1.144 (0.559–2.339)	0.713
Others	40 (10.0)	19 (12.0)			1.104 (0.432–2.818)	0.836

## Data Availability

All datasets used in this study are reasonably available from the corresponding authors.

## Abbreviations

The following abbreviations are used in this manuscript:

NMNCHC	Non-marital non-commercial heterosexual contact
NMCHC	Non-marital commercial heterosexual contact
NMHC	Non-marital heterosexual contact
TDR	Transmitted drug resistance
CRIMS	Comprehensive Response Information Management System
WHO	World Health Organization
NNRTIs	Non-nucleoside reverse transcriptase inhibitors
NRTIs	Nucleoside reverse transcriptase inhibitors
PIs	Protease inhibitors
CRFs	Circulating recombinant forms
URFs	Unclassified recombinant forms
DRMs	Drug resistance mutations
ABC	Abacavir
AZT	Zidovudine
3TC	Lamivudine
ATV/r	Atazanavir/r
DRV/r	Darunavir/r
FPV/r	Fosamprenavir/r
D4T	Stavudine
DDI	Didanosine
FTC	Emtricitabine
IDV/r	Indinavir/r
LPV/r	Lopinavir/r
SQV/r	Saquinavir/r
TDF	Tenofovir
EFV	Efavirenz
RPV	Rilpivirine
NVP	Nevirapine
NFV	Nelfinavir
TPV/r	Tipranavir/r
DOR	Doravirine
ETR	Etravirine
GD	Genetic distance
TCs	transmission clusters
INSTIs	Integrase strand transfer inhibitors
